# Australian Indigenous model of mental healthcare based on transdiagnostic cognitive–behavioural therapy co-designed with the Indigenous community: protocol for a randomised controlled trial

**DOI:** 10.1192/bjo.2020.16

**Published:** 2020-04-06

**Authors:** Maree Toombs, Bushra Nasir, Steve Kisely, Srinivas Kondalsamy-Chennakesavan, Leanne Hides, Neeraj Gill, Gavin Beccaria, Sharon Brennan-Olsen, Kayley Butten, Geoffrey Nicholson

**Affiliations:** Indigenous Health, Rural Clinical School, Faculty of Medicine, The University of Queensland, Australia; Rural Clinical School, Faculty of Medicine, The University of Queensland, Australia; Psychiatry, Faculty of Medicine, The University of Queensland, Australia; School of Psychology, Faculty of Health and Behavioural Sciences, The University of Queensland, Australia; Rural Clinical School, Faculty of Medicine, The University of Queensland; and School of Medicine, Griffith University, Australia; School of Psychology and Counselling, The University of Southern Queensland, Australia; Department of Medicine-Western Health, The University of Melbourne, Australia; Institute of Health and Biomedical Innovation and Centre for Children's Health Research, Queensland University of Technology, Australia

**Keywords:** CBT, transdiagnostic therapy, Indigenous, depressive disorders

## Abstract

**Background:**

A four- to seven-fold increase in the prevalence of current mood, anxiety, substance use and any mental disorders in Indigenous adults compared with non-Indigenous Australians has been reported. A lifetime prevalence of major depressive disorder was 23.9%. High rates of comorbid mental disorders indicated a transdiagnostic approach to treatment might be most appropriate. The effectiveness of psychological treatment for Indigenous Australians and adjunct Indigenous spiritual and cultural healing has not previously been evaluated in controlled clinical trials.

**Aims:**

This project aims to develop, deliver and evaluate the effectiveness of an Indigenous model of mental healthcare (IMMHC). Trial registration: ANZCTR Registration Number: ACTRN12618001746224 and World Health Organization Universal Trial Number: U1111-1222-5849.

**Method:**

The IMMHC will be based on transdiagnostic cognitive–behaviour therapy co-designed with the Indigenous community to ensure it is socially and culturally appropriate for Indigenous Australians. The IMMHC will be evaluated in a randomised controlled trial with 110 Indigenous adults diagnosed with a current diagnosis of depression. The primary outcome will be the severity of depression symptoms as determined by changes in Beck Depression Inventory-II score at 6 months post-intervention. Secondary outcomes include anxiety, substance use disorder and quality of life. Outcomes will be assessed at baseline, 6 months post-intervention and 12 months post-intervention.

**Results:**

The study design adheres to the Consolidated Standards of Reporting Trials (CONSORT) statement recommendations and CONSORT extensions for pilot trials. We followed the Standard Protocol Items for Randomised Trials statement recommendations in writing the trial protocol.

**Conclusions:**

This study will likely benefit participants, as well as collaborating Aboriginal Medical Services and health organisations. The transdiagnostic IMMHC has the potential to have a substantial impact on health services delivery in the Indigenous health sector.

The burden of disease attributable to mental health disparities account for as much as 16% in Indigenous Australians^1^ (in this manuscript, we respectfully refer to Aboriginal and Torres Strait Islander people as Indigenous Australians), which may be a major underestimation given accurate data on prevalence of mental disorders in Indigenous Australian have not been available. Our previous mental health prevalence study found that the prevalence of current common mental disorders in a cohort of 544 Indigenous Australians was more than four times that of the general Australian population, markedly higher than previous estimates.^2^ Standardised prevalence rates of current, 12-month and lifetime mood disorders were 16.2% (12.2–20.2%), 19.5% (15.1–23.8%) and 32.2 (27.1–37.3%), respectively; 6.4, 3.1 and 2.2 times higher, respectively, than those of the general Australian population.^3^

Although disproportionate burden of mental disorders among Indigenous people has been attributed to the intergenerational impact of colonisation, others have acknowledged that interventions and services currently available may not be effective or culturally appropriate in supporting the health needs of Indigenous Australians.^4^ Despite this notion, and the high levels of poor mental health,^2^ the efficacy of treatments has rarely been assessed in controlled trials.^5^ An urgent priority exists for the treatment of mental illness, and specifically depression, in this population. Major barriers (cultural, economic and geographic) exist to the utilisation of the limited ambulatory services available. In addition, virtually no robust evidence exists regarding effective and culturally acceptable treatments for mental disorders in Indigenous people.

## Addressing the imbalance

There is now considerable literature available on the importance of culturally appropriate healthcare^6^ and guiding principles need to achieve community acceptance and ‘buy in’ from patients.^7^ Treatments and services that have been efficacious in other health conditions, have been those that include Indigenous people in the design and delivery of the service, and recognise and support cultural concepts of health, social and emotional well-being.^8^ To facilitate an inclusive approach to healthcare development, a desirable methodology is the application of community-based participatory research (CBPR) principles and strategies.^9^ CBPR is a partnership approach to research that equitably involves community members, organisational representatives and researchers in all aspects of the research process and in which all partners contribute expertise and share decision-making and ownership.^9,10^ For instance, some studies have utilised CBPR principles and strategies to establish that land, culture, spirituality, ancestry, family and community are central to the Indigenous Australian understanding of mental health and therefore central to the design of a mental health treatment programme.^11^

For Indigenous Australians, the role of mental health and well-being revolves around holistic concepts of social and emotional well-being. The National Aboriginal and Torres Strait Islander Social Survey (NATSISS).^12^ identifies these concepts including those of cultural attachment/participation, identity, language, spirituality, loss and traditional activities. Dockery^13^ demonstrated the construct validity of the NATSISS through factor analysis. He also found strong scores of association and positive responses to questions investigating levels of happiness and mental health measures derived from the 36-item Short-Form Health Survey. Positive associations of identity and cultural attachment not only associated with happiness, but quantifiable social measures such as education, employment and general health.^13^ Indigenous conceptions of mental health are deeply rooted in the history of colonisation and cultural understandings. Further, the intersection of conceptions of ethnicity and mental illness can be detrimental to the overall social and emotional well-being of a group.^14^ Therefore incorporating social, emotional, spiritual and cultural well-being with psychological therapies is a necessary way forward to reducing the burden of mental health issues for Indigenous Australians. Given that depression is conceptualised within Western epistemology it is argued that there is no ideal measure of depression within Australian Indigenous populations.^15^ Although no specific validation measure is available, nevertheless, the Beck Depression Inventory (BDI) and the BDI-II have been used as a measure of depression in studies with both Australian and Canadian Aboriginal populations.^16^ Moreover, the BDI-II is specifically designed to be used as an outcome measure for cognitive–behaviour therapy (CBT) as it directly assesses the cognitions of the patient.

## Transdiagnostic CBT

Of the psychological therapies, CBT has the strongest evidence base for treating most mental disorders, especially depression, anxiety (including post-traumatic stress disorder) and substance use disorders.^17^ However, CBT could still be further refined to improve outcomes for patients in the treatment of comorbid mental disorders.^17^ Transdiagnostic CBT approaches to treatment that cut across diagnostic boundaries to target the common cognitive, emotional, physiological or interpersonal processes that underlie and maintain mental health symptoms may be most beneficial for individuals with comorbid disorders.^18,19^

A recent meta-analysis of transdiagnostic psychological treatments for adult depressive and anxiety disorders found they resulted in large reductions in anxiety, depression and comorbid depression and anxiety disorders, as well as moderate improvements in quality of life that were maintained for 3–6 months after treatment.^20^ Evidence for transdiagnostic approaches to comorbid substance use and mental disorders is also emerging.^21^ Research, however, is yet to explore the efficacy of either traditional or transdiagnostic CBT among Indigenous populations. A recent qualitative study supported the suitability and effectiveness of CBT for Indigenous peoples.^20^ Five university-educated Aboriginal counsellors, trained in CBT for the project, noted the adaptability of the techniques and reported they were effective in enhancing the well-being of Aboriginal clients.^20^ They also highlighted the need for CBT to be adapted to the social and cultural contexts in which it will be delivered.^20^

## Aims and objectives

The primary objective will be to determine the effectiveness of the model of care (Indigenous model of mental healthcare (IMMHC)) in Indigenous participants with depression by measuring differences between changes in the treatment as usual (TAU) and IMMHC groups on depression scores as measure by the BDI-II from baseline to 6 months.

Secondary objectives will include: evaluating the sustainability of improvements in BDI-II scores over a period of 12 months, monitoring changes in comorbid mental health conditions (any anxiety disorder and/or substance use disorder) over 12 months, and changes in quality of life, changes in subsequent healthcare service use and associated costs, and the effectiveness and appropriateness of recruitment and study procedures over the 12 months.

## Method

### Study design

The study is a randomised controlled trial (RCT) of an IMMHC, based on transdiagnostic CBT and co-designed with the Indigenous community, to evaluate the effectiveness of the treatment of depression. Trial registration: ANZCTR Registration Number: ACTRN12618001746224 and World Health Organization Universal Trial Number: U1111-1222-5849.

The development of the programme will use CBPR principles^22^ and involve community members and Elders; psychologists currently working at the Aboriginal Medical Service (AMS); psychologists from other medical services; Indigenous mental health workers; Indigenous peer-support workers; Indigenous spiritual healers; AMS board members; and current and former patients, and will be manualised upon development. Additional focus groups and one-on-one interviews will be conducted during each phase of the co-design, co-delivery and evaluation of the study. CBPR principles will ensure that a collaborative approach to the research protocol is implemented. This will determine appropriate language and the concepts that are used in the reduction of depression as described by those participating in the CBPR. It will also provide important insight into mental health treatment issues and approaches that need to be considered.

The study design adheres to the Consolidated Standards of Reporting Trials (CONSORT) statement^23^ recommendations and CONSORT extensions for pilot trials.^24^ We followed the Standard Protocol Items for Randomised Trials statement recommendations in writing the trial protocol.^25^

### Randomisation procedure

This will be a randomised controlled parallel group (1:1) trial in adult Indigenous participants with depressive disorders from participating AMSs. Participants will be randomly allocated to IMMHC or TAU (1:1 ratio) in block sizes of 2, 4, 6, 8 and 10. Block sizes will be allocated unequally using Pascal's triangle in the ratio 1:4:6:4:1. This will reduce any predictability of the randomisation codes or the blocks. Concealment will be achieved using opaque envelopes. Those who prepare the randomisation list and/or the concealed envelopes will not be involved in recruitment or data collection.

### Setting

Study activities will be conducted at locations associated with AMSs and other mainstream medical services in Toowoomba and Warwick (South East Queensland, Australia).

### Sample size calculation

The sample size estimates are based on a 2 × 2 repeated measures design comparing changes in BDI-II scores between the two groups (IMMHC and TAU). The primary goal of the study is to compare the change across time in the IMMHC group with the change across time in the TAU group. Sample sizes of 44 in both groups achieve 80% power to detect a difference in mean changes of 6.8 with a standard deviation of 12.8 at the first time point, a standard deviation of 12.0 at the second time point, and a correlation between measurement pairs of 0.6. The significance level (alpha) is a *P*-value of 0.05 using a two-sided, two-sample *t*-test. Based on previous research and experience a 20% drop-out rate is anticipated. Assuming a 20% drop-out rate, the total sample size required is 110.

### Inclusion criteria

- 18 years or older and of Aboriginal and/or Torres Strait Islander descent.
- Able to provide written informed consent.
- English-language proficiency sufficient to complete study questionnaires.
- A current (30-day) diagnosis of depression (any subtype), with or without other mood, anxiety or substance use disorders as diagnosed from a Structured Clinical Interview for DSM-5 Disorders (SCID-5)^23^ interview.

### Exclusion criteria

- Cognitive impairment, as determined through the Kimberley Indigenous Cognitive Assessment (KICA) screening.^24^
- High-risk of suicide, as determined by the SCID-5 clinical interview, and question nine on the BDI.^25^
- Recent (past 6 weeks) change in psychiatric medication.
- Any mental or physical condition requiring hospital admission.
- Diagnosis of organic brain disorder.
- Bipolar disorder.
- Psychotic disorder.
- Another mental disorder (for example likely personality disorder based on client self-report) that, in the opinion of a clinical psychologist, is likely to interfere with their ability to engage in or complete the study.
- Another medical condition likely to prevent participation in the study for 12 months.

### Participant recruitment procedure

Participants will be individuals attending participating AMSs and mainstream medical services in either Toowoomba or Warwick. Recruitment methods will include: advertising using flyers and posters, recruitment in the AMS waiting rooms and word of mouth. If individuals are interested, they will be provided a reply slip to fill out, via mail or in person, and will be given a participant information pack. An appointment for the screening interview may be made at this time if they are agreeable or will be made based on the suggested time that the participant indicates on the reply slip. The researchers will meet with the staff member/s prior to recruitment and ensure they understand the RCT sufficiently to provide the initial introduction to potential participants at the recruitment stage.

- Advertising: posters advertising the project will be displayed in the participating AMSs and medical services, and on local community noticeboards.
- Cold calling: a dedicated and trained staff member of the AMS will contact participants from our previous studies who consented to be re-contacted for future research to discuss the study. Those interested in finding out more will be posted a participant information pack.
- Waiting room recruitment: a trained staff member will recruit participants from the waiting area of the AMS or medical service. The staff will approach potential participants and provide information about the study.
- Word of mouth: past studies with this population have shown word of mouth to be an effective method of recruitment so this will be used for the current study.

### Study arms

#### Intervention – IMMHC

This will be documented and delivered by qualified psychologists trained in the treatment package. The psychologists will receive weekly cultural and clinical supervision and fidelity will be assessed by video-taping a random selection of sessions (*n* = 20) over the duration of the intervention. These tasks will be performed by the supervising clinical psychologist. Competence and fidelity to CBT will be assessed using the Cognitive Therapy Scale – Revised to rate a random selection of videotaped sessions (*n* = 20) over the duration of the entire study. Treatment will consist of both individual and group psychotherapy, as described below. Individual sessions will comprise tailored CBT and traditional spiritual healing; group sessions (co-facilitated with an indigenous health worker) will focus on skills practice, including yarning (informal conversation) about successes and difficulties in application, and developing peer support and reinforcement in this area.

- Individual treatment. The first session will involve the assessment of cognitive and behavioural factors maintaining the disorder. As our previous study^2^ demonstrated that these assessments are typically lengthy, the sessions will be up to 90 min. The treating psychologist will then develop a tailored 6-month treatment plan, in consultation with a senior clinical psychologist and the traditional healer, as well as the participant. Changes in symptomatology may warrant adjustments or changes to the treatment plan. A minimum of 10 and up to 12 sessions of individual treatments will be provided by qualified psychologists using a combination of face-to-face delivery for primary treatment and telephone delivery to facilitate skills practice. The duration of this intervention period will vary for participants, based on their tailored 6-month treatment plan.
- Group treatment. The efficacy of CBT relies on the use of ‘homework’ or skills practice by the patient to maintain treatment effects. Based on our previous research, we believe that skills practice is likely to be difficult for many Indigenous participants, so we will use traditional healing sessions as a way to do homework. Traditional healers will work with participants to find out what their homework is and practise this with individuals. In addition, follow-up calls by the traditional healers and project team to check on participants and support them between sessions will be incorporated. Group treatment will also be provided via a ‘yarning skills group’ as an adjunct to individual sessions. The purpose of this group is to build on skills learned in individual sessions, provide further peer support in a positive supportive environment, and focus on personal and group strengths. Group treatment aims to be therapeutic, strengths-based, and culturally friendly by: using traditional and spiritual healers, psychologists and Indigenous care co-coordinators as group facilitators; exploring successes in applying skills and learnings obtained from individual sessions; and allowing peers to support each other through learning new behaviours. Consistent with another evidence-based therapy that has adapted CBT for complex presentations using both individual and group CBT; a different psychologist will be used for individual and group sessions. Participants will be offered three to five group sessions over a period of 6 months, with no more than ten participants in each session group.

#### TAU

All diagnoses arising from the SCID–5 Screening interview will be entered on patient files and clients’ name provided to the AMS or medical service for follow-up. Participants who are randomised into the TAU group will receive the standard treatment offered to clients with a diagnosed mental illness. This will include a first appointment with a general practitioner who will then determine the most appropriate treatment required for the participants. This may include referral to a psychologist; a mental healthcare plan; and/or medication.

#### Post-study treatment for the control group

Should the IMMHC prove more efficacious than TAU, participating AMSs and/or medical services will adopt it as a standard treatment option for individuals diagnosed with depression. Following the intervention study, the 6-month IMMHC intervention will also be made available to research participants in the control group. It is the intention of the research team to use psychologists participating in the study where practicable, including the delivery of the IMMHC. This should ensure that at least one IMMHC trained psychologist is available at the participating AMS/medical service to deliver the model of care post-study. In addition, should the IMMHC prove more efficacious than TAU, the research team undertakes to provide a post-study training module to ensure that there are staff adequately qualified to deliver the IMMHC package into the future.

#### Interviews

All participants, from both the intervention and TAU groups, will be invited to attend individual and/or group interviews at the conclusion of the trial, after the follow-up assessments post-intervention have been completed, to discuss the reasons why participants may or may not engage with the study or choose to drop-out. Interviews will be audio-recorded for subsequent qualitative analyses.

### Outcome measures

#### Primary outcome measure

The difference between changes in the TAU and IMMHC groups on depression scores as measured by the BDI-II from baseline to 6 months.

#### Secondary outcome measures

- The difference in change of scores as measured by BDI-II from baseline to 12 months, between the TAU and IMMHC group.
- Comparison of changes in quality of life measured using the Assessment of Quality of Life (AQoL)-8D.^26^
  - Changes in quality of life between baseline and 6 months; other changes between baseline and 12 months.
- Other changes in mental health.
  - Anxiety: changes in Beck Anxiety Index (BAI^27^) – baseline to 6 months and baseline to 12 months.
  - Substance use: changes in scores based on the Alcohol, Smoking and Substance Involvement Screening Test (ASSIST).^28^
  - Changes in the presence of major depression, anxiety or substance use disorder(s), as established by a SCID-5-Clinician Version (SCID-5-CV) interview at 12 months.
- Changes in number of mental health diagnoses and patterns of psychotropic medication use.
  - Diagnostic status of initial mental health conditions will be evaluated using SCID-5-CV at 6 months and 12 months.
  - Medications (class, dosage and frequency) will be captured for both the groups and compared.
- Thematic analysis to explore reasons why participants may or may not engage with the study or choose to drop-out.

### Data analysis

Data will be gathered at the point of initial screening for eligibility, baseline, 6- and 12-month post baseline follow-ups, and at the focus groups and interviews. See the schedule of assessments in Appendix 1.

#### Quantitative analysis

Intention-to-treat principles will be used for all analyses. Differences in mean change in BDI-II scores from baseline to 6 months between the two groups will be analysed using repeated measures ANOVA, with treatment as the between-subjects factor, and time as within-subjects factor, and an interaction term (time and treatment group). Additional analyses will use mixed models adjusting for baseline covariates including quality of life. We will also calculate the differences between the groups using hierarchical mixed models for continuous variables (BDI-II and BAI scores measured at three time points – baseline, 6 months post-intervention and 12 months post-intervention) containing random intercept and random slope as well as fixed effects for treatment. We will also evaluate changes in diagnostic status using logistic and/or Poisson regression models.

#### Qualitative analysis

Thematic analysis^29^ using NVivo will be undertaken to identify recurring themes in the interview transcripts, and guide interpretation and representation of the information collected. The emerging themes will guide identification, analysis, organisation and description of the IMMHC intervention. A professional transcription service will be engaged for the transcription of the interviews.

### Ethics approval

The authors assert that all procedures contributing to this work will comply with the ethical standards of the relevant national and institutional committees on human experimentation and with the Helsinki Declaration of 1975, as revised in 2008. All procedures involving human patients were approved by the University of Queensland Human Research Ethics Committee approved the study (Clearance No: 2017001872) as well as the Board of Directors of the AMS. The study will be conducted according to National Health and Medical Research Council of Australia guidelines and the *Ethical Conduct in Research with Aboriginal and Torres Strait Islander Peoples and Communities: Guidelines for Researchers and Stakeholders*.^30^

### Strengths and limitations

For most patients, psychology-based therapy is more feasible than treatment by psychiatrist because of the chronic severe shortage of the latter in Indigenous areas. Additionally, Indigenous mental health workers, nurses and general practitioners trained in basic CBT can contribute to its delivery. Multistage evaluation will provide useful information for the planning and implementation of other Indigenous health interventions. Development of a community-designed treatment model will promote its effective national- and international-level implementation to sustain integration of translational outcomes.

## Results

**Fig. 1:**
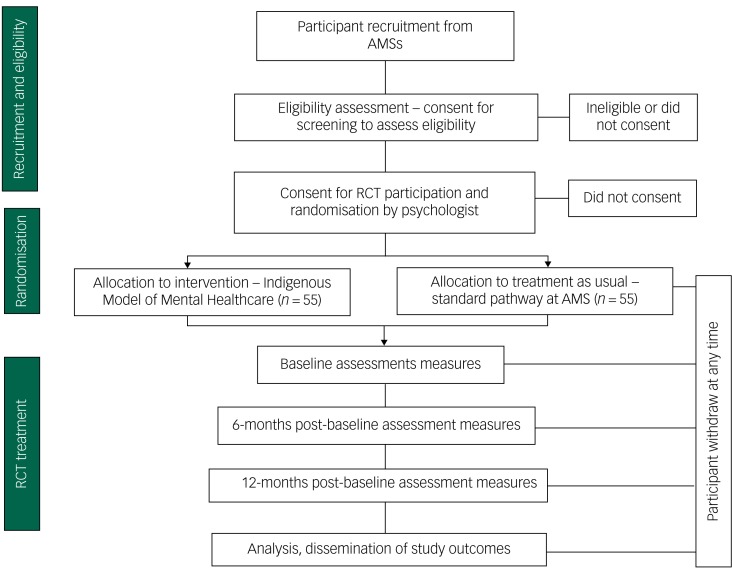
Study flow chart for the Indigenous model of mental healthcare versus treatment as usual for participants with a current 30-day diagnosis of depression.

RCT, randomised control trial; AMS, Aboriginal Medical Service.

The study design adheres to the Consolidated Standards of Reporting Trials (CONSORT) statement^31^ recommendations and CONSORT extensions for pilot trials.^32^ We followed the Standard Protocol Items for Randomised Trials statement recommendations in writing the trial protocol.^33^ See Fig. 1 for the study flow chart.

## Discussion

Mental illness is a major contributor to the Indigenous health and mortality gaps. Rectifying the adverse effects of over 200 years of colonisation by addressing the social determinants underlying this burden will be slow. In the meantime, an urgent priority exits among the population for treatment of their mental illnesses. In addition, virtually no robust evidence exists regarding efficacious and culturally acceptable treatments for mood, anxiety and substance use disorders in Indigenous Australians.

This project will address an issue of great importance to the health of Indigenous Australians. The development of an innovative, effective and sustainable treatment programme that is endorsed by the community will result in a highly significant advance in knowledge in this field and major health gains that will translate to significant changes in policy and practice. Community ‘buy in’ is an essential component to Indigenous health interventions and this project is the product of a decade of robust and meaningful community engagement. This study will be the first known controlled trial to develop an IMMHC, which assimilates both Indigenous spiritual and cultural knowledge with contemporary psychological therapies, such as CBT. This will be based on CBT, incorporating cultural and narrative elements and the concepts of social and emotional well-being.

## Data Availability

All authors all have full access to the study data.

## Supplementary material

For supplementary material accompanying this paper visit http://dx.doi.org/10.1192/bjo.2020.16. click here to view supplementary material

## Appendix

### Schedule of study assessments

**Table:**

	KICA	SCID-5	BDI-II	BAI	ASSIST	AQoL
Screening for eligibility	✓	✓	–	–	–	–
Baseline assessment	–	–	✓	✓	✓	✓
6 months from baseline	–	✓	✓	✓	✓	✓
12 months from baseline	–	✓	✓	✓	✓	✓

KICA, Kimberley Indigenous Cognitive Assessment; SCID-5, Structured Clinical Interview for DSM-5; BDI-II, Beck Depression Inventory-II; BAI, Beck Anxiety Inventory; ASSIST, Alcohol, Smoking and Substance Involvement Screening Test; AQoL, Assessment of Quality of Life.
