# Australian Indigenous model of mental healthcare based on transdiagnostic cognitive–behavioural therapy co-designed with the Indigenous community: protocol for a randomised controlled trial – CORRIGENDUM

**DOI:** 10.1192/bjo.2020.26

**Published:** 2020-04-16

**Authors:** Maree Toombs, Bushra Nasir, Steve Kisely, Srinivas Kondalsamy-Chennakesavan, Leanne Hides, Neeraj Gill, Gavin Beccaria, Sharon Brennan-Olsen, Kaley Butten, Geoffrey Nicholson

**Keywords:** CBT, transdiagnostic therapy, Indigenous, depressive disorders

This article originally referred to author Kaley Butten as ‘Kayley Butten’. This was incorrect and we apologise for the error.
